# Reconstruction of the Nose: Management of Nasal Cutaneous Defects According to Aesthetic Subunit and Defect Size. A Review

**DOI:** 10.3390/medicina56120639

**Published:** 2020-11-25

**Authors:** Luigi Losco, Alberto Bolletta, Diletta Maria Pierazzi, Davide Spadoni, Roberto Cuomo, Marco Marcasciano, Enrico Cavalieri, Ana Claudia Roxo, Paola Ciamarra, Carmen Cantisani, Emanuele Cigna

**Affiliations:** 1 Department of Translational Research and New Technologies in Medicine and Surgery, Plastic Surgery Unit, University of Pisa, 56125 Pisa, Italy; alb.bolletta@gmail.com (A.B.); dilettapierazzi@gmail.com (D.M.P.); davidespadoni@hotmail.it (D.S.); emanuele.cigna@unipi.it (E.C.); 2 Department of Medicine, Surgery and Neuroscience, Unit of Plastic and Reconstructive Surgery, University of Siena, 53100 Siena, Italy; robertocuomo@outlook.com; 3 Department of Surgery, Plastic Surgery Unit, Sapienza University of Rome, 00185 Rome, Italy; dott.marcomarcasciano@gmail.com (M.M.); cavalieri.enri@gmail.com (E.C.); 4 Plastic Surgery Unit, Pedro Ernesto University Hospital, Rio de Janeiro 20551-030, Brazil; anacwroxo@hotmail.com; 5 Dermatology Unit, Agostino Gemelli University Hospital, 00168 Rome, Italy; paolaciamarra@libero.it; 6 Dermatology Unit, Policlinico Umberto I Hospital, Sapienza Medical School, 00185 Rome, Italy; carmen.cantisani@uniroma1.it

**Keywords:** nasal reconstruction, nose reconstruction, skin cancer, nasal defect, local flap, skin graft, bilobed flap, dorsal nasal flap, nasal tip, algorithm

## Abstract

The nose represents the most common site for the presentation of cutaneous cancer, especially in sun-exposed areas: ala, dorsum, and tip. Even the smallest loss of substance can create aesthetic and psychosocial concerns for patients; therefore, surgeons who perform nasal reconstruction should be strictly confident with the pertinent surgical anatomy in order to tailor the procedure to the patient’s condition and needs. Radical tumor excision and satisfactory aesthetic and functional results are primary targets. Restoring the original shape is the goal of any reconstruction: appropriate reshaping of three-dimensional geometry, proper establishment of symmetry, and excellent color and texture match to the adjacent structures are paramount features. Multiple options exist to re-establish functional and aesthetic integrity after surgical oncology; nevertheless, the management of nasal defects can be often challenging, and the gold standard is yet to be found. The current goal is to highlight some of the more common techniques used to reconstruct cutaneous defects of the nose with a specific focus on decision making based on the aesthetic subunit and defect size. The authors attempt to share common pitfalls and offer practical suggestions that they have found helpful in their clinical experience.

## 1. Introduction

Nasal defects can result from oncologic surgery, burns, and trauma. Although this article focuses on reconstruction following tumor resection, the reviewed criteria can be effectively applied to traumatic issues; in the cases of burns and trauma, the initial management should be focused on removing any possible wound contamination before surgery. The nose and the periocular region own a key position in the facial mosaic; its peculiar anatomy combined with the functional, social, and aesthetic concerns makes its reconstruction challenging. Moreover, patients’ expectations are always higher; aesthetics becomes of paramount concern after having gone through the feelings connected with the disease [1,2,3,4].

The high prevalence of neoplasms highlights the assumption that the nose is one of the most projecting and sun-exposed facial units, bearing an increased risk for melanoma and, more frequently, nonmelanoma skin cancers [5,6,7]. Surgery still represents the gold standard approach for skin cancer treatment and may be combined with Mohs micrographic surgery; anyway, a conservative treatment must be preferred before a histopathologic examination for malignant lesions has been verified [8,9].

Nasal defects can involve the skin, cartilage, and bone, or the internal mucosal lining, in various degrees, and even the smallest loss of substance can create aesthetic and psychosocial concerns for patients. Therefore, surgeons who perform nasal reconstruction should be strictly confident with the pertinent surgical anatomy in order to tailor the procedure to the patient’s needs [10].

The first technical description of nasal reconstruction was made by Indian physicians thousands of years ago [11]. Since then, many refinements have been made to these primitive procedures, and many novel techniques have been reported. Burget and Menick [12] have further improved nasal reconstruction techniques by establishing the aesthetic subunit principle. Locoregional flaps and skin grafts still play a significant role in the reconstruction of soft tissue and cutaneous defects [13]; anyway, microsurgery can offer alternative options, especially for total or subtotal nasal reconstruction.

The current goal is to highlight some of the more common techniques used to reconstruct cutaneous defects of the nose with a specific focus on decision making based on aesthetic subunit and defect size. The authors attempt to share common pitfalls and offer practical suggestions that they have found helpful in their clinical experience.

## 2. Defect Evaluation

Nasal tissue may be divided into cover (skin, subcutaneous tissue, and muscle), framework (cartilage and bones), and internal lining (vestibular skin and nasal mucosa). The key for a successful nasal reconstruction is a careful evaluation of the defect and nearby tissue status (i.e., investigate any primary or secondary tumors or scars). The most critical aspects to investigate are the size, depth, and location of the defect [14].

Generally, small defects of <1.5 cm can be closed primarily or reconstructed with a flap (local tissue) or full thickness skin graft (FTSG). Medium defects of 1.5–2.5 cm can be reconstructed with a flap (regional or local tissue) or FTSG. When the defects are large, i.e., >2.5 cm, a flap (regional tissue) or FTSG should be exploited. However, inconsistent recommendations for a general ranking are found in the literature, and disagreements exist about whether the cut off for a primary closure should be 1 cm instead of 1.5 cm or whether a regional flap is mandatory for a medium-sized defect [15,16].

A variability of ±0.5 cm should be applied to the previous and any subsequent classification in order to take into account any difference in size and skin mobility among patients; a pinch test has to be completed preoperatively, especially when performing a primary closure or a local flap.

In our practice, we do not select any defect for secondary intention healing because the aesthetic result is likely to be unacceptable. Secondary healing requires weeks of careful wound care and, often, social segregation; surgical site infection or unpleasant scarring are likely, although the literature is inconclusive about this matter [17,18]. Indeed, the small scars that result from second intention healing located in natural concavities (i.e., alar grooves) are often less evident than the bulkiness of flaps and grafts; anyway, secondary healing is much more unpredictable than primary healing, and a subsequent alar elevation would be an aesthetic disaster.

Microvascular free flaps are the gold standard in head and neck reconstruction, and they have proven to be highly reliable and effective in total and subtotal nasal reconstruction; however, those techniques are beyond the scope of the present review [19,20,21,22,23].

Nose reconstruction is the perfect example of how reconstructive surgery can be tailored to a patient’s characteristics/comorbidities and expectations. It is mandatory to have a truthful talk with patients when discussing the optimal modality among those in the surgeon’s armamentarium. A staged procedure should be avoided in elderly patients with multiple comorbidities; moreover, in patients who are active smokers, or have only recently quit, it is advisable to avoid skin grafts when possible and perform minimal thinning of any skin flap. These patients should be counseled about their increased risk for complications.

## 3. The Nose: A Split Unit

**Figure 1:**
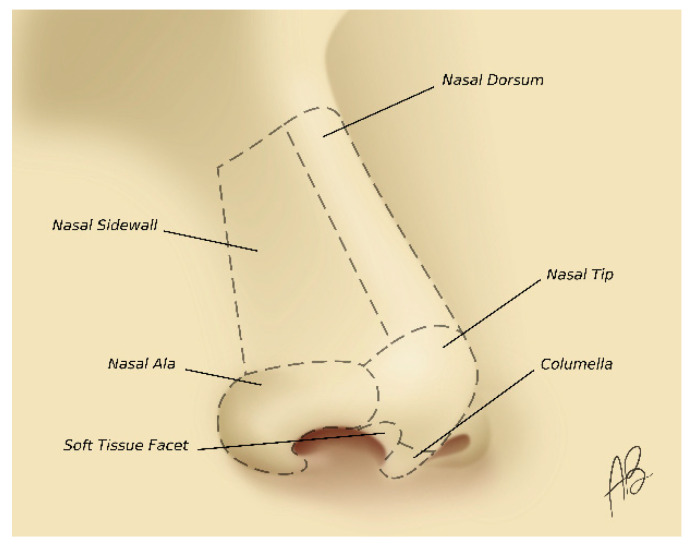
Aesthetic subunits of the nose.

The nose is one of the aesthetic units of the face and is subdivided into nine aesthetic subunits. They are identified by distinctive convex or concave surfaces, more specifically, from cranial to caudal third as dorsum and paired sidewalls, nasal tip and paired alae, columella, and paired soft tissue facets [24] (Figure 1). Cutaneous nasal defect reconstruction should be considered in light of nasal aesthetic subunit principles. If most of the surface area (>50%) of a convex subunit (tip or ala) is excised, reconstructing the whole subunit is usually preferable: the safe part of the subunit should be excised and the whole subunit should be reconstructed [25,26]. Beyond the aesthetic purpose of this widespread principle, it has to be kept in mind that an inadequate resection determines severe consequences in terms of recurrence rate. Following this method, the incisions lie at the joint of nasal subunits along natural boundaries, which enhances the symmetry and provides concealment of scars. Further, an expected subtle scar contraction will produce a mild elevation of the subunit, which enhances the convex nature of the tip or ala unit [27,28].

The subunits differ in skin quality and shape, and the latter is determined by the size and contour of the underlying structural framework. The nasal tip, nasal alae, and cranial dorsum (radix) have the thickest skin with more pilosebaceous units. Conversely, the skin of the mid-lower dorsum and the upper nasal sidewall is usually the thinnest. Reconstructive surgeons should be conscious of these differences because the recruitment of skin with a thickness that differs from that of the recipient area may lead to suboptimal results; moreover, a thick and non-elastic skin interferes with flap transposition by increasing wound tension and developing conspicuous standing cutaneous deformities.

## 4. Patients and Methods

All the patients signed an informed consent form and a photo release form. Perioperative antibiotics such as azithromycin are administered in nasal reconstruction cases. We inject carbocaine and ropivacaine with epinephrine in the subfascial (SMAS) plane (also in the eventual flap donor area). The same protocol is used in the case of a patient undergoing general anesthesia with the aim of preventing massive and bothersome bleeding during surgery.

When facing a nasal cutaneous defect, we never choose healing by secondary intention because the outcomes are unpredictable in terms of cosmetic results. In our practice, local flap reconstruction is generally chosen over skin grafts, although the latter can be beneficial in (1) patients who reject any staged procedure and/or multiple flap harvest, (2) patients with superficial defects, and (3) patients with significant medical comorbidities; anyway, a higher necrosis rate should be taken into account if compared to local flap surgery, and a suboptimal result should be expected in terms of contour and color match. The surgical wound is covered with an appropriate dressing in order to minimize infections and bleeding; for the same purpose, patients are also advised to keep the head elevated in the early postoperative period. All procedures performed were in accordance with the 1964 Helsinki declaration and its later amendments or comparable ethical standards.

## 5. Management of Defects

Loss of substance affecting the nose can be classified according to the location. The authors will offer a subunit-based classification of the reconstructive armamentarium highlighting the pearls and pitfalls of the inherent techniques (Table 1). Most of the techniques described in the following section can be performed under local anesthesia. We inject carbocaine and ropivacaine with epinephrine in the subfascial (SMAS) plane (also the eventual flap donor area). The same protocol is used in the case of a patient undergoing general anesthesia with the aim of preventing massive and bothersome bleeding during surgery. Defects involving loss of framework or mucosa require the replacement of skeletal support and lining following the principle of replacing like-with-like [29].

### 5.1. Skin Grafting

Skin grafting is always an option for the reconstructive surgeon, although a suboptimal result should be expected in terms of contour and color match. However, the contour could be acceptable if repairing the thin skin of dorsum and sidewalls; on the other hand, the outcome could be unsatisfactory if dealing with extremely thick and sebaceous skin. Full-thickness skin grafts are suitable for reconstructing most superficial defects; however, the color-blending could be often disappointing, especially in sun-damaged reddish skin, where the FTSG will appear as a pale patch.

**Figure 2:**
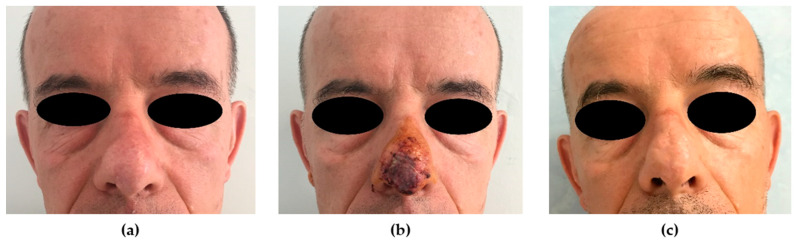
Skin graft reconstruction. (**a**) Preoperative picture. A 45-year-old patient was affected by morphea-like basal cell carcinoma (BCC)of the tip of the nose. Although the patient was advised about the suboptimal result offered by a skin graft reconstruction, he refused any scar on the forehead. (**b**) An FTSG was planned and harvested from the preauricular area (best skin thickness match with the tip of the nose) to reconstruct the 2.5 × 2.3 cm skin defect. (**c**) The 12-month postoperative picture shows an acceptable result in terms of color and contour match; a slightly depressed area can be noticed on the midline.

When facing a large multi-subunit defect, the surgeon should be encouraged to adopt the well-recognized concept of reconstructing the nose in aesthetic subunits. Using the subunit principle is often ideal [30]; however, it could be demanding and require the harvest of two or more flaps. Although local flap reconstruction is generally chosen over skin grafts, the latter can be beneficial in patients who reject any staged procedure, have superficial defects, or have significant medical comorbidities (Figure 2). A higher necrosis rate should be taken into account if compared to local flap surgery.

Considerations in choosing a donor site include the size of the required graft, the thickness and grade of sun exposure of the donor and recipient site skin, and the resultant cosmetic deformity/easy concealment of the donor site. Preferred donor sites include preauricular, postauricular, and supraclavicular areas and the melolabial fold [14,31]. When a large graft is necessary and a color match is suitable, the authors prefer to exploit the inner arm area (as per brachioplasty) at the proximal middle-third; this site is more concealable than the supraclavicular area.

Dermabrasion can help level areas with a rough texture or slightly irregular contour and can help blend areas with a color mismatch. It can enhance the final outcome in patients treated with full-thickness skin grafts and can be exploited when a smooth transition is required between local flap and native tissue. When needed, dermabrasion is usually performed three months after the surgical procedure.

#### Pearls and Pitfalls

FTSG exploited for tip reconstruction should be thinned minimally due to the characteristics of caudal nose skin

## 6. Dorsum and Sidewalls

The dorsum and sidewalls are treated together because these two aesthetic subunits are similar in terms of skin characteristics and reconstructive options. The dorsal and sidewall skin is usually mobile and less sebaceous. Local tissue can be easily mobilized to reconstruct small- to medium-sized defects; instead, flaps from adjacent areas are exploited for large defects.

**Figure 3:**
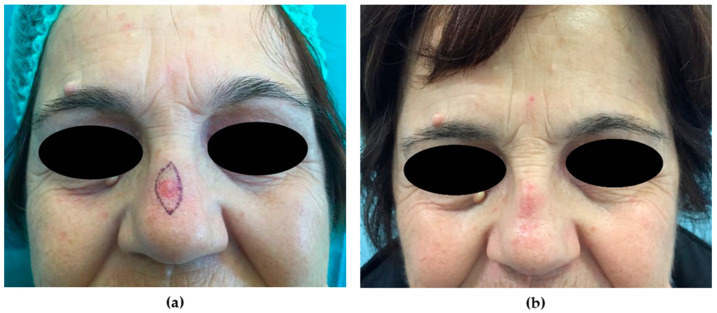
Primary closure. (**a**) Preoperative picture. A 57-year-old patient was affected by BCC of the dorsum of the nose. A primary closure repair was planned due to the small size of the skin defect (0.9 cm, intraoperatively); a pinch test was accomplished to evaluate skin redundancy. (**b**) Six-month postoperative picture shows a fair result.

**Figure 4:**
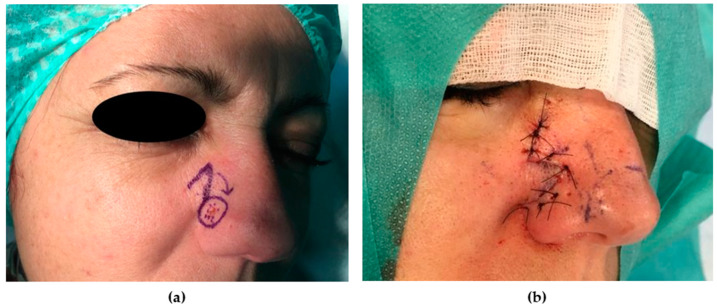
Transposition flap. (**a**) Preoperative picture. A 39-year-old patient was affected by BCC of the right nasal sidewall. A transposition flap was planned due to the small size of the skin defect (1.2 cm, intraoperatively); a pinch test was accomplished to evaluate skin mobility. (**b**) Intraoperative picture. After the flap was transposed and the surrounding skin was widely undermined, the skin was sutured.

**Small defects** of <1.5 cm could be closed primarily due to the slight redundancy of cutaneous tissues. In the dorsum, the decision of whether to orient direct closure in the transverse or craniocaudal dimension should be made according to the shape and size of the defect (Figure 3). Another reconstructive option is the glabellar flap. For sidewall repair, a transposition flap, typically a note flap, is better suited (Figure 4). A line is drawn tangent to the defect and 1.5 times the diameter of the defect, and then a triangular flap is completed by designing another line with an angle of 50–60° [32].

**Figure 5:**
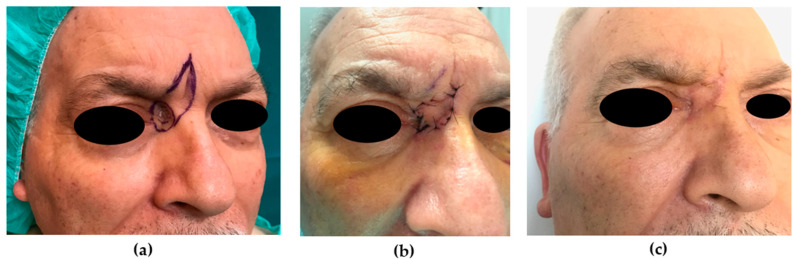
Glabellar flap. (**a**) Preoperative picture. A 67-year-old patient was affected by BCC of the right nasal sidewall. A V-Y advancement glabellar flap was planned (skin defect 1.4 cm). (**b**) Intraoperative picture. After the flap was advanced on a subcutaneous pedicle, the skin was sutured in V-Y fashion. (**c**) Nine-month postoperative picture shows a good result.

**Medium defects** of 1.5–2.5 cm are not suitable for a primary closure. A local flap reconstruction is mandatory. In the cephalic dorsum (and sidewalls), a glabellar flap can mobilize a conspicuous amount of skin and cover a defect extending to the canthus. The flap can be harvested as a V-Y advancement flap (Figure 5) or a more typical glabellar rotation flap.

**Figure 6:**
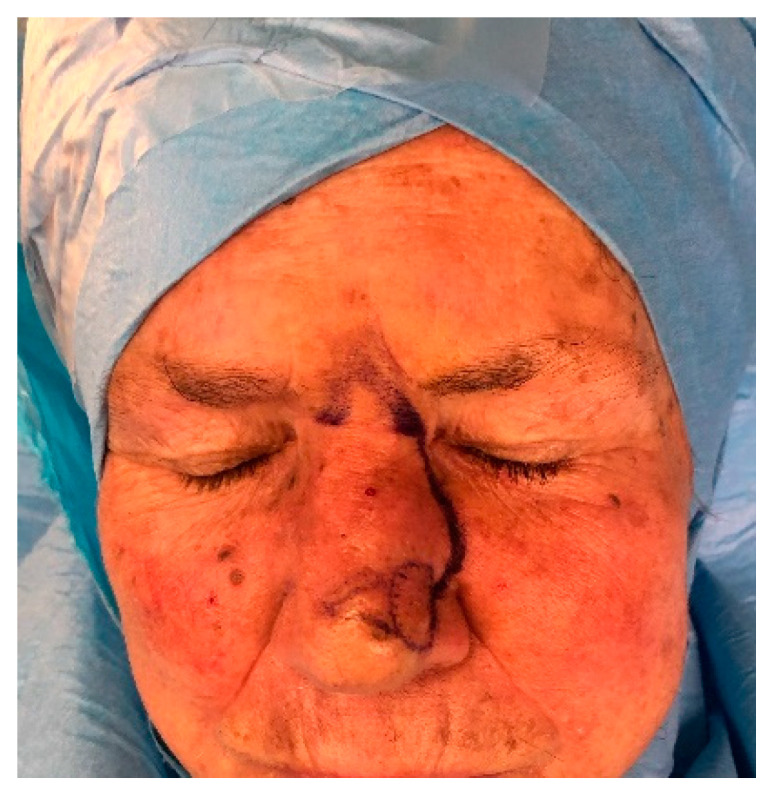
Dorsal nasal flap, preoperative markings. A “V” shape was designed in an upside-down fashion over the glabella; then, a curvilinear line was drawn on the nasal–cheek junction till reaching the defect site. On the same side of the pivot area, a standing cutaneous deformity will be evident after the flap has rotated. It can be drawn preoperatively, as shown; anyway, the authors suggest to double-check it intraoperatively.

**Figure 7:**
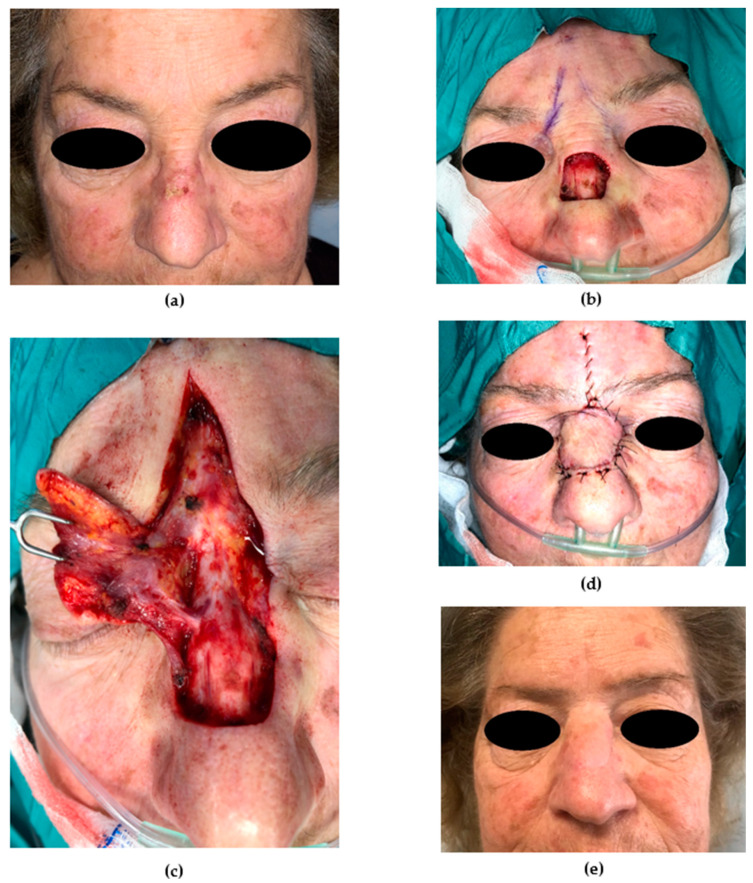
Dorsal nasal flap. (**a**) Preoperative picture. A 71-year-old patient was affected by BCC of the dorsum of the nose. (**b**) A dorsal nasal flap was planned to repair the skin defect (2.2 × 1.8 cm); previously, a pinch test was carried out to assess the amount of skin redundancy. (**c**) An axial pattern dorsal nasal flap was sculpted; the vascular supply was provided by a branch of the angular artery. The glabellar portion of the flap was elevated in the subcutaneous plane, and the nasal portion was harvested beneath the muscular plane. (**d**) The flap could be rotated, minimizing the standing cutaneous deformity. The donor site defect was closed primarily. (**e**) The 12-month postoperative picture shows an optimal result in terms of color and contour match.

In the middle–caudal dorsum, a bilobed flap is a good reconstructive option because it can recruit the right amount of skin from the sidewalls; however, the dorsal nasal flap is the authors’ choice. It is an “extended” glabellar flap that recruits the glabellar redundancy in a V-Y fashion along with the majority of nasal skin (cranial to the defect). The dorsal nasal flap as originally described by Rieger [33] is a laterally based rotation flap. A “V” is designed in an upside-down fashion over the glabella, and then a curvilinear line is drawn on the nasal–cheek junction till it reaches the defect site (Figure 6). Basically, Rieger described a useful random rotation flap with a wide pedicle. Recently, Marchac and Toth [34] refined Rieger’s flap by narrowing the pedicle and depicting an axial flap based on the vessel arising from the inner canthus. This allowed increased mobility, precise adjustment, and less wound closure tension (Figure 7).

A dorsal nasal flap can be used also for **large-sized defects** of >2.5 cm located on the caudal dorsum–tip of the nose. However, in those cases, a paramedian forehead flap should be preferred to obtain a more acceptable aesthetic outcome. Cheek advancement may be performed, but the aesthetic result would be suboptimal due to the obliteration of the nasofacial sulcus. Additional procedures are often required to restore the nasofacial groove and normal external contour of the nose [27].

### Pearls and Pitfalls

- If a direct closure is planned, a careful and extensive undermining should be always accomplished in order to avoid any standing cutaneous deformity and wound closure tension.
- In the caudal dorsum, a transverse fusiform excision should be reserved for the elderly in order to obtain a pleasing tip rotation (balancing nasal tip ptosis); if otherwise exploited, such direct closure could cause a tip distortion.
- If a thickness mismatch is present (i.e., glabellar skin moved to the inner canthus), thinning of the flap is mandatory in order to obtain a pleasing aesthetic result.
- Transposition flaps should be reserved for the reconstruction of these subunits. In the caudal nose, the thicker skin is inelastic; as a result, any transposition flap would generate conspicuous standing cutaneous deformity and excessive wound closure tension.

## 7. Nasal Tip

The reconstruction of this subunit could be tough and peculiar due to the intrinsic features of the area (i.e., its convexity and the sebaceous and firm skin). The indication for primary closure can be given only for rather small defects of 0.5 cm located on the midline after a pinch test is done; it is necessary to evaluate the skin excess and mobility with the aim to prevent any slight alar elevation.

**Small defects** of <1.5 cm could be treated with a bilobed flap. This flap should be the favorite option in reconstructing circular defects on the caudal nose. The thick sebaceous skin prevents any attempt to sculpt a classic transposition flap; furthermore, the potential distortion of adjacent structures (i.e., alar rim) necessitates the recruitment of skin from nasal sidewall and dorsum, where it is more abundant and pliable. A bilobed flap (a double transposition flap with a single base) with its geometrical features recruits elastic skin from contiguous subunits and minimizes the wound closure tension and standing cutaneous deformities [35]. The bilobed flap can be based laterally or medially.

**Figure 8:**
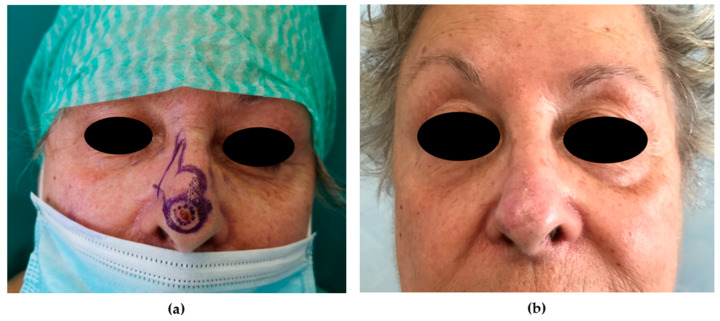
Bilobed flap. (**a**) Preoperative picture. A 64-year-old patient was affected by BCC of the nasal tip. A laterally based bilobed flap was planned to repair the skin defect (1.4 cm). The surface area and the width of the first lobe should be the same as the defect. The second lobe was designed in a triangular shape; the width was slightly less and the height was approximately 1.5 times greater than the first lobe. (**b**) The 12-month postoperative picture shows an optimal result in terms of color and contour match.

The first lobe (curvilinear) is designed immediately adjacent to the defect. The surface area and the width of the first lobe should be the same as the defect. The second lobe should have a surface area smaller than that of the defect and should be designed in a triangular shape; the width should be slightly less and the height should be approximately 1.5 times greater than the first lobe. The straight axes passing through the center of each lobe and defect are placed at about 45° from each other. The dissection is carried out below the muscular plane and above the perichondrium/periosteum. As for any other transposition flap, the donor site of the second lobe is closed first. Then, the first lobe is transferred to reconstruct the nasal defect. Lastly, the second lobe is trimmed and stitched in the donor defect of the first lobe, and the standing cutaneous deformity (SCD) is excised. The removal of the SCD has to take into account the position of the nasal ala in order to avoid its cranial displacement (Figure 8).

**Medium defects** of 1.5–2.5 cm can be reconstructed with a dorsal nasal flap. A common downside, especially in >2 cm defects, could be the cranial displacement of the tip (elevation of ala if the defect is not located on the midline). A paramedian forehead flap (PFF) will usually give a more natural result because the entire aesthetic unit can be restored and the scars can be concealed in natural boundaries. Anyway, a dorsal nasal flap is still preferred if a staged procedure is refused by the patient.

**Large defects** of >2.5 cm are commonly reconstructed with a PFF. The forehead flap is based on the supratrochlear vessels that pierce the orbital bone at about 2 cm lateral to the midline. It is commonly used for total resurfacing of the tip, nasal dorsum, and sidewalls or ala. The procedure can also be used for reconstruction of multiple subunits. It is a staged operation; two-stage reconstruction is more commonly performed, but it also can be performed in three stages in order to reduce complications and enhance the aesthetic outcomes [36,37,38].

### Pearls and Pitfalls

When using a bilobed flap, issues may arise with standing cutaneous deformities (SCDs). Some authors mark SCDs preoperatively; we prefer to do it intraoperatively because the amount of skin to be excised depends on multiple factors such as skin thickness, skin redundancy, surface area of the defect, and the angles between the lobes (if exceeding 100°, the SCD may be more relevant) [39].

- The thick sebaceous skin of the caudal nose tends to invert even if the suture is correctly completed. We perform a dermo-epidermal interrupted suture in order to achieve wound edge eversion.
- A bilobed flap should be drawn at least 0.5 cm away from the nostril margin to obtain a pleasing result and avoid deformities.
- The curvilinear incisions of the bilobed flap increase the risk of developing a trap-door deformity. A wide undermining of the flap and nasal skin reaching the nasal–cheek boundaries should be accomplished to hinder it. Another key maneuver that could help the surgeon to prevent the deformity is adjusting the thickness of the flap; anyway, it should be accomplished while avoiding any vascular impairment.

## 8. Nasal Ala

The alar anatomy is peculiar. The majority of malignancies involving alar skin also require the removal of the fibrofatty subdermal tissue that gives structure and contour to the ala. Indeed, cartilage grafts are often exploited when facing alar defects in order to prevent obstruction of the airway and upward migration of the ala [40].

Superficial defects could be treated with a full-thickness skin graft and a cartilage alar rim graft in order to prevent nostril distortion. In case of deeper defects, a complete resurfacing of the ala is recommended, so we will not provide any size-based algorithm. The medial cheek is the preferred source for flap harvesting.

A nasolabial transposition flap is a viable option for alar reconstruction; anyway, this single-stage option results in a deformation of the alar facial sulcus (if left intact after tumor removal) and loss of symmetry that has to be restored with subsequent poor cosmetic result [41,42].

An interpolated melolabial flap does not retain such downsides, and it is a recommended option if the alar facial sulcus was left intact after tumor excision. This two-stage procedure leaves the alar facial sulcus untouched without the need for subsequent reconstruction [24,31]. The main drawback of this flap is a cosmetic deformity that lasts three weeks; a second procedure is necessary to divide the flap pedicle and inset the flap. Defects also affecting the alar facial sulcus and/or alar groove often require a third stage during which these natural boundaries are accurately recreated.

In a young, thin patient without redundant skin on the midface, a PFF could be preferred [31].

### 8.1. Full-Thickness Defects

**Figure 9:**
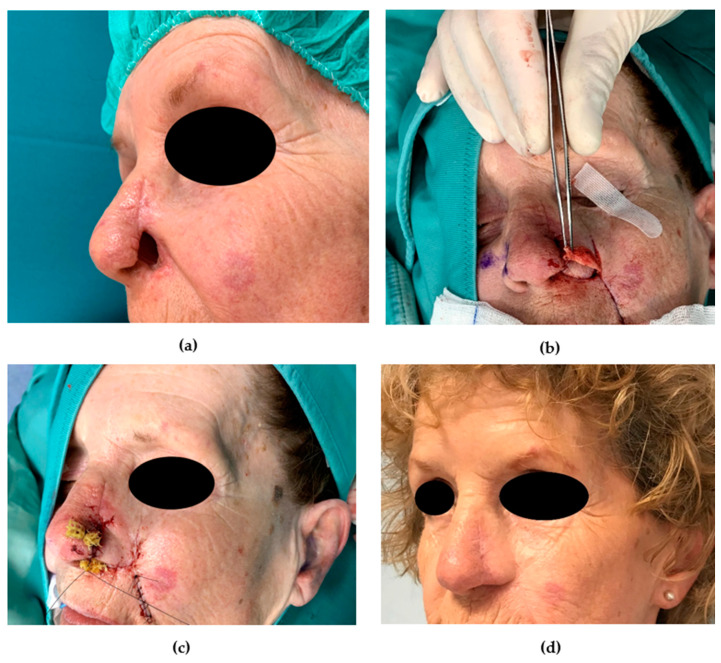
Nasolabial folded flap. (**a**) Preoperative picture. A 68-year-old patient was affected by melanoma of the nasal ala. A nasolabial folded flap was planned to repair the full-thickness defect in two stages. (**b**) A nasolabial flap was folded to reconstruct the lining and external coverage after being thinned. A cartilage graft (held by the forceps) was harvested from the left auricle and molded to provide structural support to the ala. (**c**) Stage I. Immediate postoperative picture. A povidone iodine impregnated gauze was positioned. The donor site was sutured primarily. (**d**) The 12-month postoperative (stage II) picture shows an optimal result; the nasal ala regained a good contour, and alar facial sulcus was restored.

Especially in alar reconstruction, due to the absence of a structural framework, the reconstructive surgeon should be able to deal with full-thickness reconstruction. Full-thickness nasal defects are challenging due to multiple layer reconstruction. For lining a total alar reconstruction, nasal septal mucoperichondrial hinge flap is a frequently used technique; however, the vascularization of mucosal flaps may be weak in active smokers, and folded PFF is an alternative technique. In both these reconstructive strategies, three or more stages are necessary for lining, cutaneous covering, and alar facial sulcus reconstruction. In patients with thin skin that refuse a PFF reconstruction, we recommend the reconstruction with a nasolabial folded flap in two stages. It has been advocated that the technique of folding a flap on itself should not be used in the internal nasal valve and nostril region because tissue bulk can obstruct the airway [10]. On the other hand, this technique provides consistent vascular supply, enables a single flap harvest with minimal social segregation, and avoids forehead scars; we perform the nasolabial folded flap in patients with thin cheek skin to reconstruct the whole ala, achieving good functional and aesthetic results (Figure 9).

#### Pearls and Pitfalls

- We suggest that during tumor removal, a paraffin gauze should be inserted in the nostril with the aim of making neoplasm removal easier and avoiding full-thickness perforation of the ala, which is likely due to the lack of a proper framework.
- If a total alar resurfacing is needed, a 1–2 mm strip of nasal skin at the alar facial junction should be preserved, if possible, in order to avoid a challenging reconstruction of this natural crease.

### 8.2. Cartilage Grafting

Cartilage grafting is mandatory in the case of the structural framework being impaired by oncological excision; it can also be used to support the soft tissues and prevent a contour deformity due to wound healing forces [14]. Alar defects should be always grafted in order to provide structural support and prevent any functional or aesthetic drawbacks. 

The grafts are usually harvested from the medial aspect of the auricle; the straight contour of the septal cartilage makes it a secondary option. The costochondral cartilage could be exploited if larger amounts of cartilage are required [43]. Grafts should be secured with absorbable monofilament suture tied in a horizontal mattress fashion with the knots placed superficial to the cartilage graft.

## 9. Postoperative Care and Complications

Perioperative antibiotics such as azithromycin should be administered in nasal reconstruction cases, especially given the proximity of the flap to colonized areas (i.e., nasal mucosal lining, nasopharynx, and oropharynx) [5].

Proper wound dressing is mandatory to avoid bleeding and minimize the chance of surgical site infection [44,45,46]. In the postoperative period, the patient could be unable to wear glasses due to the bandage size; this should be disclosed during the preoperative visit. Postoperative bleeding is not a rare complication due to the considerable facial vascularization, especially in patients taking antiplatelet drugs. In case a hematoma is noticed, it should be promptly treated in order to avoid any compromise to graft or flap viability. Facial and especially nasal vascularization is extremely well represented, and the flaps can have a lower width–length ratio than other areas of the body; anyway, flap necrosis and distal skin tip necrosis may occur if (1) the flap is overly thinned, (2) the pedicle base is made too narrow, or (3) the arc of rotation is made too sharp when a flap is folded over the defect.

Another common problem following flap reconstruction of nasal defects is trap-door deformity. This complication often occurs when a circular scar contracts during wound healing causing elevation of the flap that results in a pin-cushion appearance. Alternatively, trap-door deformity can result from (1) inadequate undermining of surrounding nasal skin, (2) hematoma formation, or (3) impairment of lymphatic drainage with subsequent lymphedema; however, the incidence of lymphedema is not easily evaluated, as seen in breast-cancer-related lymphedema or lower limb lymphedema [47,48].

A subtle trap-door deformity does not require any specific treatment besides frequent massaging; this should confirm the lymphatic impairment behind the etiology of this complication [49,50]. The second-line conservative treatment is triamcinolone injections. Trap-door deformity that does not respond to conservative measures can be addressed surgically. A portion of the scar is reopened, and the flap is elevated in the subdermal plane, avoiding elevation of the scar attached to deeper structures. The scar and deep tissue are then accurately excised/molded with a blade or scissor to achieve a satisfactory contour. The skin is precisely trimmed to fit the defect [14].

### Pearls and Pitfalls

If surgery is performed under local anesthesia, we recommend cooperating with the anesthesiologist; the surgery can be longer than an hour, and any patient could feel discomfort with people touching his face: slight sedation may help. Furthermore, any rise in blood pressure could result in exaggerated intraoperative bleeding.

- We suggest that the patient should keep the head elevated in the early postoperative period if bleeding is expected (i.e., sit on a chair, sleep with two pillows). Applying ice for the same purpose should be done with caution after a local flap is harvested. Indeed, if the cold source is placed over the pedicle (right after the surgery), it could impair flap vascularization.
- If a local flap reconstruction is performed in anticoagulated patients, we usually schedule an outpatient visit on the day after surgery to detect any possible hematoma and prevent any subtle flap ischemia.

## 10. Conclusions

Nasal reconstruction is deeply founded on the nasal subunit principle, and the physician must bear in mind the various tissue quality among the subunits when outlining a reconstructive strategy. Primary targets of nasal reconstruction are complete tumor resection along with a satisfactory cosmetic and functional result. Reconstructive surgeons should treat each patient as a distinct individual with a peculiar defect, and the procedure should be customized according to the clinical conditions and the demands and wishes of our patients.

## Figures and Tables

**Table 1:** Defects and reconstructive strategies.

Aesthetic Subunit	Technique *
Dorsum and Sidewalls, cm	
<1.5	Primary closure Transposition flap (sidewall) Glabellar flap
1.5–2.5	Glabellar flap (cranial defect) Bilobed flap Dorsal nasal flap
>2.5	PFF Dorsal nasal flap ** Cheek advancement flap
Tip, cm	
<0.5	Primary closure
<1.5	Bilobed flap V-Y island pedicle advancement flap
1.5–2.5	Dorsal nasal flap ** PFF
>2.5	PFF
Ala ***	Nasolabial flap Melolabial interpolated flap PFF

PFF: Paramedian forehead flap. * FTSG was not mentioned because it can be applied virtually to any subunit, see text. ** Consider aesthetic result. *** In case of deeper defects, a complete resurfacing of the ala is recommended, see text.
